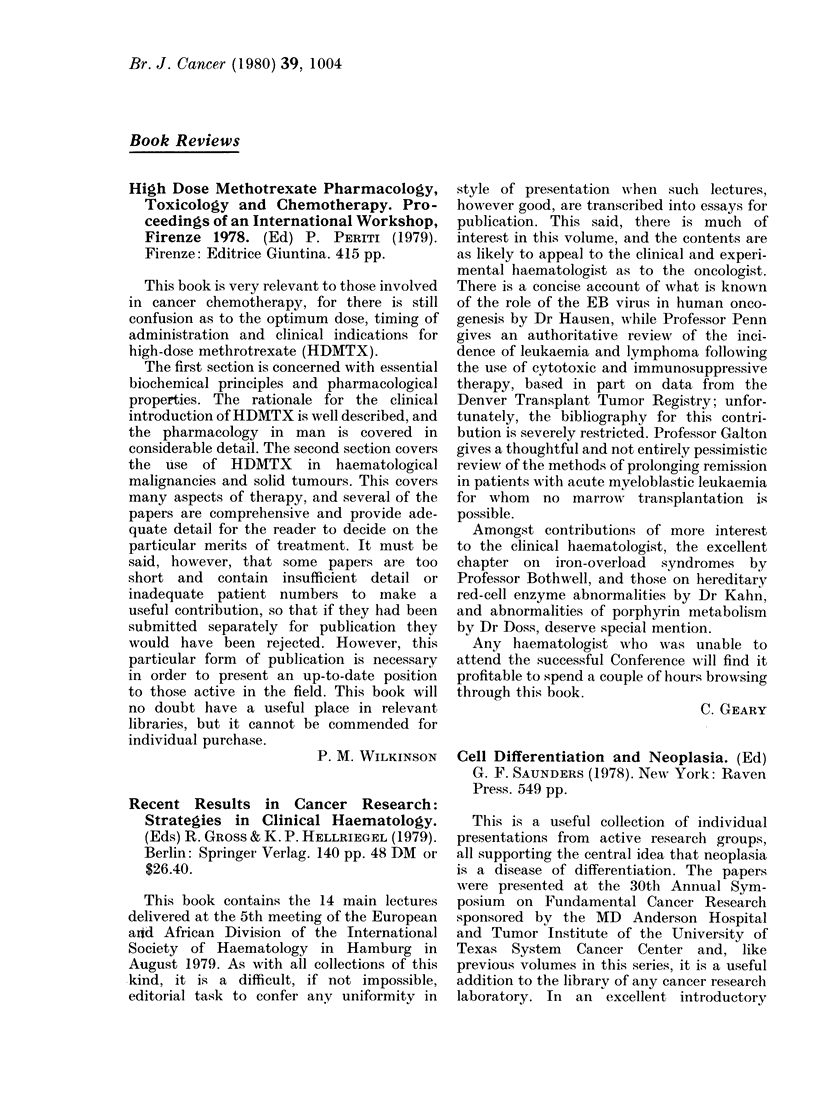# Recent Results in Cancer Research: Strategies in Clinical Haematology

**Published:** 1980-06

**Authors:** C. Geary


**Recent Results in Cancer Research: Strategies in Clinical Haematology.** (Eds) R. Gross & K. P. Hellriegel (1979). Berlin: Springer Verlag. 140 pp. 48 DM or $26.40.

This book contains the 14 main lectures delivered at the 5th meeting of the European and African Division of the International Society of Haematology in Hamburg in August 1979. As with all collections of this kind, it is a difficult, if not impossible, editorial task to confer any uniformity in style of presentation when such lectures, however good, are transcribed into essays for publication. This said, there is much of interest in this volume, and the contents are as likely to appeal to the clinical and experimental haematologist as to the oncologist. There is a concise account of what is known of the role of the EB virus in human oncogenesis by Dr Hausen, while Professor Penn gives an authoritative review of the incidence of leukaemia and lymphoma following the use of cytotoxic and immunosuppressive therapy, based in part on data from the Denver Transplant Tumor Registry; unfortunately, the bibliography for this contribution is severely restricted. Professor Galton gives a thoughtful and not entirely pessimistic review of the methods of prolonging remission in patients with acute myeloblastic leukaemia for whom no marrow transplantation is possible.

Amongst contributions of more interest to the clinical haematologist, the excellent chapter on iron-overload syndromes by Professor Bothwell, and those on hereditary red-cell enzyme abnormalities by Dr Kahn, and abnormalities of porphyrin metabolism by Dr Doss, deserve special mention.

Any haematologist who was unable to attend the successful Conference will find it profitable to spend a couple of hours browsing through this book.

C. Geary